# Individual Participant Data Meta‐Analysis Identifies Risk Factors for Acute and Persistent Posttraumatic Stress Disorder and Depression Symptoms Following Trauma

**DOI:** 10.1155/da/9859948

**Published:** 2026-05-04

**Authors:** Andrew Ratanatharathorn, Richard A. Bryant, Ronald C. Kessler, Katherine M. Keyes, Yutaka Matsuoka, Miranda Olff, Renato Polimanti, Andrew G. Rundle, Karestan C. Koenen, Arieh Y. Shalev

**Affiliations:** ^1^ Department of Epidemiology, TH Chan School of Public Health Harvard University, Boston, Massachusetts, USA, harvard.edu; ^2^ School of Psychology, University of New South Wales, Sydney, New South Wales, Australia, unsw.edu.au; ^3^ Black Dog Institute, Sydney, Australia; ^4^ Department of Health Care Policy, Harvard Medical School, Boston, Massachusetts, USA, harvard.edu; ^5^ Department of Epidemiology, Mailman School of Public Health Columbia University, New York, New York, USA, columbia.edu; ^6^ National Cancer Center Japan Institute for Cancer Control, Tokyo, Japan; ^7^ Department of Psychiatry, Amsterdam University Medical Centers, University of Amsterdam, Amsterdam Public Health, Amsterdam Neuroscience, Amsterdam, Netherlands, uva.nl; ^8^ ARQ National Psychotrauma Centre, Diemen, Netherlands; ^9^ Department of Psychiatry, Yale University School of Medicine, New Haven, Connecticut, USA, yale.edu; ^10^ Broad Institute of MIT and Harvard, Boston, USA, broadinstitute.org; ^11^ Department of Psychiatry, Massachusetts General Hospital, Boston, Massachusetts, USA, harvard.edu; ^12^ Department of Psychiatry, Hebrew University of Jerusalem, Jerusalem, Israel, huji.ac.il; ^13^ Department of Psychiatry, NYU Grossman School of Medicine, New York, New York, USA, med.nyu.edu

## Abstract

**Background:**

Posttraumatic stress disorder (PTSD) and major depressive disorder (MDD) are common sequelae to trauma. Identifying individuals at risk for persistent symptomology allows for targeted interventions.

**Methods:**

We conducted an individual participant data meta‐analysis (IPD‐MA) by pooling data from five prospective emergency room and critical care unit studies (*N* participants = 2571) to examine risk factors for PTSD and MDD. We derived harmonized measures of PTSD and MDD in the acute period (0–60 days) following a traumatic event and in the follow‐up persistent period (122–456 days). Multinomial logistic regression was used to estimate associations between seven risk factors for acute and persistent MDD and PTSD. Logistic models examined the association between acute symptoms and persistence of PTSD and MDD.

**Results:**

Female sex (odds ratio [OR] range: 1.48–2.14) was associated with increased risk for acute and persistent MDD and PTSD while individuals who experienced an accident versus an assault or other traumatic event as the index trauma were at reduced risk (OR range: 0.39–0.66). Acute MDD symptom severity was associated with persistent PTSD (OR: 1.17; 95% CI: 1.08, 1.27) and remained significant after inclusion of acute PTSD symptom severity (OR: 1.14; 95% CI: 1.01, 1.29). In an analysis of PTSD symptom clusters, reexperiencing symptoms (OR: 1.18; 95% CI: 1.02, 1.36) and MDD symptom severity were associated with persistent PTSD. In models of persistent MDD, acute PTSD symptom severity was associated with persistence (OR: 1.15; 95% CI: 1.03, 1.28), but neither overall symptom severity nor cluster symptom severities were associated with persistence after inclusion of acute MDD symptom severity (OR: 1.13; 95% CI: 1.00, 1.26).

**Conclusion:**

Early symptoms of MDD and reexperiencing were associated with the persistence of psychopathology indicating that depressive rumination may play a role in the maintenance of MDD and PTSD.

## 1. Introduction

Seventy percent of people world‐wide will experience at least one traumatic event during their lifetime [1, 2]. In the United States alone, over 42 million individuals visit emergency departments each year after experiencing a traumatic injury [3]. Two common psychological sequelae of trauma are posttraumatic stress disorder (PTSD) and major depressive disorder (MDD) [4–6], which are also highly comorbid with 52% of individuals with PTSD reporting comorbid MDD [7]. PTSD and MDD are also associated with the development of chronic diseases such as cardiovascular disease [8–11] and diabetes [12–14] as well as mortality [10, 15, 16]. Prior research has found that 73% of individuals with PTSD symptoms and 61% with MDD symptoms report remission of symptoms in the years following experiencing a traumatic event [17], leaving a substantial proportion with persistent psychopathology. Early interventions are effective in preventing post‐trauma psychopathology, however they are resource intensive [18–20]. Identifying individuals at risk for persistent symptoms would allow limited resources to be used more effectively during months following trauma when it is unknown whether an individual is at risk for persistent symptoms or will naturally remit [21, 22]. Specifically, with reliable identification of risk groups, individuals at high risk of persistent symptomology could be targeted for nonpharmacologic treatments (e.g., cognitive behavior therapy), which have fewer adverse effects and are more efficacious than pharmacologic interventions (e.g., antidepressant agents [19, 23]) but are more expensive [18–20].

While previous studies found that PTSD and MDD share several risk factors, including experiencing childhood abuse [24, 25], lower socioeconomics status [24, 26, 27], and social isolation [24, 28, 29], these studies have at least two limitations. First, most risk factor research has focused on current cases, which are a combination of persistent and incident cases making it impossible to differentiate between a risk factor that increases the risk of incidence and one that increases the risk of persistence. This distinction is critical in post‐trauma psychopathology as many individuals have high levels of PTSD and/or MDD symptoms shortly after trauma and may even qualify for a diagnosis but for most individuals these symptoms remit on their own while a smaller proportion have persistent problems that require intervention [17]. Identifying risk factors for acute onset and persistence following trauma can indicate when interventions targeting specific subgroups would yield the greatest results in preventing the development of acute or persistent PTSD or MDD. For example, the approximately two‐fold higher prevalence of MDD in women compared to men is driven by women’s higher incidence of MDD, not by differences in course as after onset men and women show similar likelihood of symptom persistence [30, 31].

Second, previous studies focus on lifetime cases, which reflect incidence but are limited by retrospective recall. For retrospective studies, recall bias may lead to underreporting of onset of psychopathology. For example, Moffit et al. [32] found that past year depression prevalence in the prospective Dunedin Multidisciplinary Health and Development Study matched those from a national retrospective study, the National Comorbidity Study Replication, but that lifetime MDD was substantially higher in the prospective study (44%) than that of the retrospective study (25%). These results can be reconciled if retrospectively assessed participants underreported past MDD symptoms, decreasing the estimated lifetime prevalence and indicating that prospective studies more accurately capture incidence [32, 33].

Beyond these limited findings, no systematic examination exists of what is associated with acute onset and persistence of PTSD and MDD following trauma. We addressed these limitations by conducting an individual participant data meta‐analysis (IPD‐MA) within the International Consortium to Predict PTSD (ICPP), an individual participant data study that pooled data from five longitudinal emergency room studies of PTSD and depression symptoms [34]. Similarly to traditional meta‐analysis, IPD‐MA combines published and unpublished studies to answer research questions [35]. However rather than combining effect sizes from published studies, IPD‐MA combines and analyzes raw data from multiple studies, which in addition to increasing power from larger sample sizes, allows for greater freedom and consistency in statistical modeling including how to address potential confounders [36], imputation of missing data including systematically missing risk factors [37, 38], and examining differences across studies [35, 39].

While previous results from the ICPP found that the strongest predictor of persistent PTSD symptoms was acute PTSD symptoms [40], unexplored in this analysis was the role that MDD symptoms have and whether individuals with acute depression following trauma later develop PTSD, as acute symptom severity for both disorders may also play a role in persistence for one or both disorders [40, 41]. We extend previous work by including MDD as an additional outcome following trauma and the interplay between acute symptoms of both disorders with their persistence to identify risk factors for psychopathology.

## 2. Methods

The literature review, data collection, and harmonization of the 13 longitudinal acute‐care–based studies of recent trauma survivors contributing to the ICPP have been previously described [34]. For this IPD‐MA, studies were required to have assessments of MDD and PTSD within 60 days of experiencing the incident trauma, referred to as the acute period. Persistent MDD and PTSD were defined as those whose diagnoses did not remit between four‐ and 15‐months following trauma (122–456 days). Five studies of the 13 ICPP studies met these criteria including the multisite acute stress disorder study (multisite ASD) [42], the Hadassah startle study [43], the Jerusalem Trauma Outreach and Prevention study (JTOPS) [22], the Tachikawa Cohort of Motor Vehicle Accident study (TCOM) [44], and the Amsterdam cortisol study (Table 1) [45]. All the studies except TCOM recruited patients in the emergency room following trauma, while TCOM recruited from a critical care unit following a motor vehicle accident. Incident traumas varied across studies. ER studies recruited patients who presented to the ER due to variety of trauma types, including motor vehicle accidents, other accidents, assault, or possible terrorist attacks [22], while TCOM only recruited victims of motor vehicle accidents [46]. All contributing studies obtained informed consent using procedures approved by their local institutional review boards.

**Table 1:** ICPP studies included in the individual participant data meta‐analysis with assessments of posttraumatic stress disorder (PTSD) and major depressive disorder (MDD) in both the acute (0–60 days since trauma) and persistent periods (122–456 days since trauma).

Study	Country	Instrument		*N*	Setting	Mean (standard deviation) days since trauma for		Follow‐up rate (%)
		PTSD	MDD			Acute assessment	Persistent assessment	
Multisite ASD [42]	Australia	CAPS	HADS	1082	ER	6.8 (7.1)	366.8 (46.7)	76.4
Hadassah startle [43]	Israel	CAPS	BDI	118	ER	37.6 (9.2)	153.3 (38.2)	87.3
JTOPS [22]	Israel	CAPS	BDI	735	ER	19.4 (5.2)	201.5 (77.0)	72.0
TCOM [44]	Japan	CAPS	HADS	155	CCU	38.9 (6.1)	201.0 (28.5)	59.4
Amsterdam cortisol [45]	The Netherlands	CAPS	HADS	481	ER	42.1 (8.6)	327.0 (95.4)	72.6

Abbreviations: ASD, acute stress disorder; BDI, Beck Depression Inventory II; CCU, critical care unit; ER, emergency room; HADS, Hospital Anxiety and Depression Scale; JTOPS, Jerusalem Trauma Outreach and Prevention Study; MDD, major depressive disorder; PTSD, posttraumatic stress disorder; TCOM, Tachikawa Cohort of Motor Vehicle Accident study.

## 3. Measures

### 3.1. PTSD

All five studies assessed PTSD using the Clinician Administered PTSD Scale (CAPS) for DSM‐IV [47, 48], PTSD symptom severity was calculated by summing the frequency and severity for all 17 PTSD symptoms yielding a continuous score from 0 to 136. Symptom severities for criterion B (reexperiencing symptoms; items 1–5), criterion C (avoidance symptoms; items 6–12), and criterion D (hyperarousal symptoms; items 13–17) were calculated by summing frequency and severity for each criterion’s items. A diagnosis of PTSD was determined following DSM‐IV criteria with participants assumed to have been exposed to a traumatic event (criterion A) as they presented to an emergency room for treatment, and endorsement of at least one re‐experiencing, three avoidance, and two hyperarousal symptoms [47]. For criteria B–D, symptoms were considered present if a frequency score of one or more and an intensity score of two or more were endorsed [48–50]. Since information on criterion E (symptoms duration of at least 1 month) and F (clinically significant distress or impairment) were not collected across all studies, E and F criteria were not included in the determination of persistent PTSD, but previous analyses have shown high concordance between case definitions with and without criteria E and F [51]. Each participant was classified into the following categories: (1) “No Diagnosis” if no diagnosis of PTSD in either time point, (2) “Acute Onset” if diagnosis in the acute time point but not during follow‐up, and (3) “Persistent” if diagnosis in both time points.

### 3.2. Depression

Across the studies, two depression instruments, the Beck Depression Inventory‐II [52] (BDI) and the Hospital Anxiety and Depression Scale [53] (HADS), were used to assess depressive symptoms. The BDI assesses all nine depression symptoms using 21 questions, while the HADS assesses only four depression symptoms using seven questions, which stems from the HADS development as an instrument for use in primary care settings where patients might be experiencing depression’s somatic symptoms due to primary illnesses rather than depression [54]. Both instruments assess the two core depression symptoms “depressed mood most of the day” and “diminished interest or pleasure in all or most activities” as well as “agitation or psychomotor retardation noticed by others” and “feelings of worthless or excessive guilt.” The BDI and HADS assess each depression symptom on a four‐point Likert scale (item range: 0–3). For example, the core depression symptom “diminished interest or pleasure in all or most activities” is assessed in the BDI by the question asking about “loss of pleasure” with possible responses of “I get as much pleasure as I ever did from the things I enjoy,” “I don’t enjoy things as much as I used to,” “I get very little pleasure from the things I used to enjoy,” “I can’t get any pleasure from the things I used to enjoy.” The HADS assesses the same symptom with the prompt “I still enjoy the things I used to enjoy:” with possible responses “Definitely as much,” “Not quite as much,” “Only a little,” and “Hardly at all.”

### 3.3. Baseline Risk Factors

We examined seven risk factors consistently found to be associated with either PTSD or depression in previous studies including sex, younger age, lower educational attainment, not being married or cohabitating have each been found to be associated with lifetime PTSD and MDD, and having experienced traumatic events prior to the index trauma [24, 55, 56]. Variables for each risk factor were extracted from ICPP studies and harmonized according to previously documented algorithms [51]. Briefly, due to differences in educational systems across countries (e.g., secondary education ends after 10 years in Australia compared with 12 in Japan of which only nine are compulsory), educational attainment across studies was dichotomized into whether or not a participant completed secondary education or not. Marital status was harmonized across studies into two categories: “Single/not‐cohabitating with a partner” and “Married/cohabitating with a partner.” While all individuals in ICPP experienced a traumatic event, type of event has been shown to be associated with both PTSD and MDD [1, 57]. The index trauma that brought individuals into an emergency room and inclusion in the study were categorized into three categories: motor vehicle accidents, other accidents, and interpersonal trauma. Prior exposures to traumatic events were harmonized by categorizing each trauma type reported as either an interpersonal (e.g., intimate partner violence, combat) or non‐interpersonal trauma (e.g., accidents). The prior interpersonal versus non‐interpersonal categories were chosen as interpersonal traumas have been associated with increased risk for PTSD and MDD compared to non‐interpersonal traumas [1, 58]. Participants who did not report any prior traumatic events were classified as having experienced no traumatic events prior to the index trauma.

## 4. Statistical Analyses

### 4.1. Equating HADS Symptom Severity to BDI Symptom Severity

Confirmatory factor analyses of the BDI and HADS have found that two‐factor models best fit both instruments [59], but with differing underlying factors due to the difference in symptom measurement. For BDI, the underlying factors represent a cognitive‐affective aspect, which encompasses the core depression symptoms, and a somatic‐vegetative factor, which encompasses the somatic symptoms (although agitation has been found in one confirmatory factor analysis of the BDI to be part of the cognitive‐affective factor) [60, 61]. For the HADS, the two factors identified map onto depression and anxiety broadly rather than specific aspects of depression [62]. Because of these differences, we harmonized depression measures across studies using item response theory (IRT) to equate depression symptom severity scores and probable depression diagnoses across the two instruments, using three PTSD symptoms that overlap with depression as anchor items [47, 63]. Supporting Information 1: Table S1 summarizes the BDI, HADS depression, and CAPS items included and their mapping to the nine DSM‐IV depression symptoms.

First, within each study the IRT assumption that the two instruments were sufficiently unidimensional was assessed using confirmatory factor analysis to test the fit of a one‐factor model. If the comparative fit index was above 0.95 [65, 66], the Tucker–Lewis Index above 0.95 [65, 66], and the root mean square error of approximation below 0.06 (or not above 0.10, indicating a poor fit) [64, 66], unidimensionality was considered confirmed. Next, two pooled datasets were created by combining studies that assessed MDD with the BDI and with HADS, in addition to the three overlapping MDD symptoms from the CAPS. To test whether the underlying construct of depression was the same between the HADS and BDI, within the combined instrument datasets two‐factor models splitting depression symptoms into somatic and non‐somatic categories based on Elhai et al. [67] were run to assess whether each instrument is assessing the same underlying factor structure of depression. Improvements on the one factor fit indicate that the two instruments are measuring the same underlying construct.

In the pooled dataset, equated BDI scores were created by fitting a graded response model to estimate the linking constants that were then applied to transform HADS scores into the BDI using the mean/sigma approach and CAPS items assessing depression symptoms (C4, D1, and D3) used as anchor items [68, 69]. This procedure yielded for each possible HADS depression score (0–21) an equated BDI total score (0–63). Table 2 shows the correspondence between scores. HADS scores were equated to BDI scores as the BDI assesses all nine DSM depressive symptoms. Our primary dichotomous depression outcome used the established BDI threshold of 20, which reflects at least moderate symptom severity and is commonly used as a cut‐off for probable MDD. Participants with BDI ≥ 20 were classified as having probable MDD. In HADS studies, we identified the HADS score equating to BDI = 20; this corresponded to HADS = 13, so participants with HADS ≥ 13 were classified as having probable MDD. Lower HADS cut‐offs (e.g., ≥7 or ≥11) mapped to BDI scores below 20 in our equating table, indicating milder depression and yielding a noncomparable case definition. Anchoring the dichotomous outcome to the BDI moderate‐severity threshold and its HADS‐equivalent score, therefore, ensured a similar level of depression severity across cohorts. Each participant was classified into the following categories: (1) “No Diagnosis” if no diagnosis of MDD in either time point, (2) “Acute Onset” if diagnosis in the acute time point but not during follow‐up, and (3) “Persistent” if diagnosis in both time points.

**Table 2:** Equated Beck Depression Inventory II (BDI) symptom severity based on Hospital Anxiety and Depression Scale (HADS) symptom severity following item response theory equating.

Original HADS score	Equated BDI score
0	0
1	1
2	3
3	4
4	5
5	7
6	8
7	10
8	11
9	13
10	15
11	16
12	18
13	20
14	22
15	24
16	27
17	30
18	33
19	37
20	42
21	63

### 4.2. ICPP Participant Characteristics by PTSD and MDD Status

Differences in frequency and severity of risk factors between participants without acute or persistent symptoms or lost to follow‐up were assessed using *t*‐tests for continuous risk factors and *χ* ^2^tests for categorical risk factors.

Multiple imputation using chained equations (MICE) was performed to impute missing data using multilevel models to account for the structure of the data where observations (level 1) at each time point are nested within individuals (level 2) [70]. Data were imputed in the combined sample with dummy variables for each study to model fixed study effects. Continuous variables were imputed using a random effects model assuming heteroskedasticity for the errors. Dichotomous variables were imputed using a probit link function rather than a logistic link as the conditional distributions of the random effects coefficients can be more easily simulated using a probit link [71]. Categorical variables with more than two levels were split into a series of dichotomous variables, which were then imputed as dichotomous variables. Imputation models for each variable consisted of variables correlated at least 0.10 with the index variable [70]. For the analysis, results from each imputed dataset were pooled using Rubin’s method [72].

### 4.3. Estimating Associations Between Risk Factors and PTSD and MDD

To assess differences in risk for acute versus persistent diagnoses, multinomial logistic models were fit separately for PTSD and MDD predicting persistent and acute onset versus the no diagnosis group (reference) using all risk factors. Differences in associations for acute onset and persistence of each disorder were tested by fitting a series of multinomial logistic regressions that constrained each risk factor in turn to be equal across outcomes, and a likelihood ratio test was performed comparing the model fit of the constrained versus the unconstrained model, where the risk factor could vary by outcome. A significant difference in model fit implied that the risk factor’s effect varied across outcomes. This is necessary to assess whether risk factors affect outcomes (e.g., acute onset versus persistent) as a null effect for one outcome and a significant effect for the other is insufficient [73].

To determine whether risk factors affect risk for persistence of symptoms after acute onset, among individuals with acute (reference group = 0) and persistent symptoms (outcome group = 1), associations between risk factors persistence of symptoms were estimated in a series of separate logistic regressions for PTSD and MDD. First, baseline risk factors and the other disorder’s acute symptom severity were used to predict a persistent diagnosis (Model 1). That is, a model was fit to predict persistent PTSD using baseline risk factors and acute depression symptoms and acute PTSD symptoms were used to predict persistent MDD. Second, as PTSD and MDD are correlated and acute symptoms of one disorder may be a proxy for the other, separate models for persistent PTSD and MDD were fit using acute symptoms of both disorders to predict persistence adjusting for all other risk factors (Model 2). Third, to examine whether specific PTSD clusters are associated with persistent PTSD and MDD, a final set of models was fit using depression symptom severity and the PTSD reexperiencing, avoidance, and hyperarousal criteria severities to predict persistence (Model 3).

To improve interpretability, we expressed odds ratios (ORs) per 5‐point increase for MDD symptom scores and PTSD symptom cluster scores, and per 15‐point increase for total PTSD severity as these increments represent meaningful changes.

### 4.4. Sensitivity Analyses

Just as effect heterogeneity due to study differences is a concern when combining studies in a traditional meta‐analysis, in an IPD‐MA estimated effects between exposures and outcomes may differ across studies due to differences in participant recruitment, follow‐up times, or other unknown factors due to heterogeneity in the pooled sample. Three analyses were conducted to assess whether study heterogeneity substantially affects each model’s results. First, between‐study heterogeneity was assessed by comparing the fits of fixed and random effects models using a bootstrap approach. Participants from the pooled sample were sampled with replacement, fixed and random effects models fit, and predicted probabilities of each PTSD and MDD outcome were estimated for the left‐out participants. Two fit statistics were estimated for each model and for PTSD and MDD. First, the ratio of estimated outcome to the actual number within each outcome (expected/observed ratio) was calculated, where a ratio above 1 indicates that the estimated prevalence of an outcome is too high and below 1 too low [39]. Second, we calculated the Brier score [74], which is the mean standard error of the squared difference between the estimated probabilities of a persistent diagnosis and actual diagnosis, to determine overall model accuracy. This process was repeated 1000 times within each imputed dataset and statistics averaged across iterations for PTSD and MDD separately. In the case that the fit indices indicate that the studies are too heterogeneous to be combined, analyses were conducted dropping each study in turn to identify a set of studies exists that are sufficiently homogeneous (e.g., good expected/observed ratio and low Brier score) to be pooled.

The persistent period was defined as 122–456 days after trauma, which could span almost a whole year difference between those at the earliest time point and latest time point. To test whether associations differ by follow‐up time, a second sensitivity analysis was conducted whereby analyses were repeated restricting the data to participants assessed between 270 and 456 days (9–15 months). Differences between the results for the full follow‐up time frame (122–456 days) and the restricted time frame (270–456) would indicate that persistent psychopathology does not emerge until more than 4 months after trauma.

### 4.5. Results

Results from confirmatory factor analysis examining the sufficiently unidimensional and factor structure of the depression measures within each ICPP study are presented in Supporting Information 1: Table S2. In no individual study was depression unidimensional, but when studies that assessed MDD with the same instrument are pooled, all indices met prespecified thresholds (HADS studies CFI = 0.99, TLI = 0.98, RMSEA < 0.10; BDI studies CFI = 0.98, TLI = 0.98, RMSEA < 0.10). Confirmatory factor analyses specifying a two‐factor model showed an improved fit for both the HADS (CFI = 0.99, TLI = 0.98, RMSEA = 0.05) and BDI studies (CFI = 0.99, TLI = 0.98, RMSEA = 0.06).

Table 2 presents the equated BDI symptom severity for each level of HADS symptom severity. Participants with a HADS symptom severity of 13 were equated to a BDI severity of 20, which is the cutoff from having a probable diagnosis of moderate depression [59].

Distributions of risk factors and baseline symptoms are presented in Tables 3 and 4. Of the 2571 participants, 803 (31.2%) were missing a PTSD assessment while 1143 (44.5%) were missing an assessment of MDD. These participants’ PTSD and MDD statuses were imputed using MICE. Participants with acute or persistent PTSD or depression were around 2 years younger than participants without a PTSD (*p* = 0.002) or MDD diagnosis (*p* = 0.019). Only 31.3% of participants classified as never having a diagnosis of PTSD were female compared to 50.9% of those classified as having an acute diagnosis or 55.8% with a persistent diagnosis (*p*  < 0.001). Similarly, 36.2% of participants classified as never having a diagnosis of MDD were female compared to 50.0% of those with an acute diagnosis and 46.5% with a persistent diagnosis. Participants who experienced an assaultive index trauma were more likely to have a diagnosis of persistent PTSD (14.6%) or acute PTSD (10.9%) compared to never having a PTSD diagnosis (4.9%) with a similar pattern seen for persistent MDD (14.3%), acute MDD (11.4%), and never MDD (5.2%). Participants with persistent PTSD reported higher number of PTSD symptoms in the acute period than those who only had an acute diagnosis (mean symptoms: 77.4 versus 64.8) and higher MDD symptoms in the acute period as well (mean symptoms: 24.1 versus 18.4). Participants with persistent MDD also reported higher PTSD symptoms (mean: 72.5 versus 60.4) and MDD symptoms (mean: 30.7 versus 25.7) compared to those with only an acute diagnosis of MDD.

**Table 3:** Distribution of baseline risk factors and acute symptom severity by PTSD status.

Variable	Never	Acute	Persistent	Missing	Total	*p*
*N*	1363 (53%)	267 (10.4%)	138 (5.4%)	803 (31.2%)	2571 (100%)	—
Age, mean (SD)	39.4 (14.5)	37.2 (12.8)	37.6 (12.4)	37.3 (13.29)	38.4 (13.9)	0.002
Female, *N* (%)	424 (31.3%)	136 (50.9%)	77 (55.8%)	283 (35.3%)	920 (35.9%)	<0.001
Single, *N* (%)	628 (48.3%)	121 (49.0%)	61 (47.7%)	378 (52.4%)	1188 (49.6%)	0.356
< Secondary education, *N* (%)	260 (20.1%)	34 (13.8%)	25 (19.7%)	150 (21.0%)	469 (19.7%)	0.092
Prior trauma, *N* (%)						0.002
Prior non‐interpersonal trauma	471 (34.7%)	84 (34.9%)	45 (35.7%)	252 (33.2%)	852 (34.3%)	
Prior interpersonal trauma	666 (49.0%)	90 (37.3%)	57 (45.2%)	364 (48.0%)	1177 (47.4%)	
Index trauma, *N* (%)						<0.001
MVA	951 (70.2%)	214 (80.5%)	110 (80.3%)	586 (73.1%)	1861 (72.7%)	
Other accidents	338 (24.9%)	23 (8.6%)	7 (5.1%)	149 (18.6%)	517 (20.2%)	
Assaults (intentional harm)	66 (4.9%)	29 (10.9%)	20 (14.6%)	67 (8.4%)	182 (7.1%)	
Baseline PTSD severity, mean (SD)	16.5 (13.0)	64.8 (15.5)	77.4 (17.9)	33.3 (25.9)	30.0 (26.2)	<0.001
Baseline depression severity, mean (SD)	5.9 (6.1)	18.4 (8.8)	24.1 (10.2)	10.7 (9.8)	9.5 (9.4)	<0.001

*Note:* Differences across categories assessed using *t*‐tests for continuous risk factors and *χ* ^2^ tests for categorical risk factors.

Abbreviations: MVA, motor vehicle accidents; PTSD, posttraumatic stress disorder.

**Table 4:** Distribution of baseline risk factors and acute symptom severity by depression status.

Variable	Never	Acute	Persistent	Missing	Total	*p*
*N*	1217 (47.3%)	140 (5.4%)	71 (2.8%)	1143 (44.5%)	2571 (100%)	—
Age, mean (SD)	39.3 (14.3)	36.5 (12.3)	37.7 (12.6)	37.8 (13.7)	38.4 (13.9)	0.019
Female, *N* (%)	439 (36.2%)	70 (50.0%)	33 (46.5%)	378 (33.2%)	920 (35.9%)	<0.001
Single, *N* (%)	622 (52.2%)	68 (48.9%)	34 (50.7%)	484 (48.4%)	1208 (50.4%)	0.357
< Secondary education, *N* (%)	209 (17.6%)	30 (21.9%)	14 (21.5%)	216 (21.8%)	469 (19.7%)	0.082
Prior trauma, *N* (%)						0.078
Prior non‐interpersonal trauma	432 (35.8%)	34 (25.0%)	24 (34.8%)	362 (33.8%)	852 (34.3%)	
Prior interpersonal trauma	553 (45.8%)	66 (48.5%)	34 (49.3%)	524 (48.9%)	1177 (47.4%)	
Index trauma, *N* (%)						<0.001
MVA	884 (73.0%)	110 (78.6%)	57 (81.4%)	810 (71.1%)	1861 (72.7%)	
Other accidents	264 (21.8%)	14 (10.0%)	3 (4.3%)	236 (20.7%)	517 (20.2%)	
Assaults (intentional harm)	63 (5.2%)	16 (11.4%)	10 (14.3%)	93 (8.2%)	182 (7.1%)	
Baseline PTSD severity, mean (SD)	22.2 (19.8)	60.4 (25.7)	72.5 (25.5)	32.2 (26.8)	30.0 (26.2)	<0.001
Baseline depression severity, mean (SD)	6.235 (5.349)	25.714 (5.377)	30.746 (8.439)	9.800 (9.400)	9.516 (9.389)	<0.001

*Note:* Differences across categories assessed using *t*‐tests for continuous risk factors and *χ* ^2^ tests for categorical risk factors.

Abbreviations: MVA, motor vehicle accidents; PTSD, posttraumatic stress disorder.

**Figure 1:**
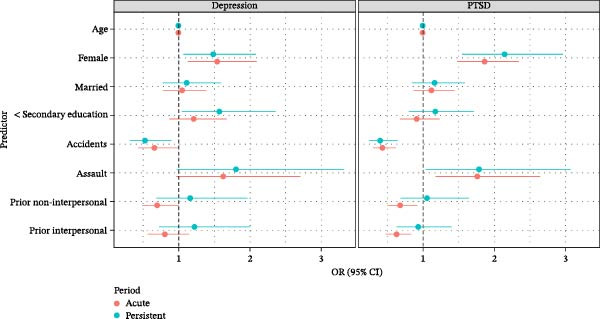
Odds ratios (OR) and 95% confidence intervals (CI) between each risk factor and acute and persistent depression or PTSD.

Associations between each predictor and acute and persistent MDD and PTSD are presented in Figure 1. Female sex was associated with increased risk of both acute (OR: 1.54; 95% CI: 1.13, 2.09) and persistent MDD (OR: 1.48; 95% CI: 1.06, 2.08) as well as for acute (OR: 1.86; 95% CI: 1.48, 2.35) and persistent PTSD (OR: 2.14; 95% CI: 1.55, 2.96). Participants who experienced an accident as the index traumatic event were less likely to develop either acute or persistent MDD and PTSD (OR range: 0.39–0.66), while participants who experienced an assault were more likely to develop acute PTSD (OR: 1.76; 95% CI: 1.17, 2.64) and persistent PTSD (OR: 1.78; 95% CI: 1.04, 3.07). Participants who reported experiencing prior non‐interpersonal (OR: 0.68; 95% CI: 0.50, 0.92) or prior interpersonal trauma (OR: 0.63; 95% CI: 0.47, 0.84) were less likely to develop acute PTSD without prior trauma.

Associations between baseline risk factors and acute symptoms with persistent MDD and PTSD are presented in Tables 5 and 6. No baseline risk factors were significantly associated with persistent PTSD or MDD when accounting for acute symptoms. In models for persistent PTSD, acute MDD symptoms were associated with persistence (Model 1: OR for a 5‐point increase in MDD symptoms: 1.17; 95% CI: 1.08, 1.27) and remained significantly associated with persistent PTSD after including acute PTSD symptoms (Model 2: OR for a 15‐point increase in PTSD symptoms: 1.14; 95% CI: 1.01, 1.29). When individual PTSD symptom cluster severities were included in the model, acute MDD symptom severity remained significantly associated with persistent PTSD (Model 3: OR: 1.14; 95% CI: 1.04, 1.25) as was acute PTSD reexperiencing symptoms (Model 3: OR for a 5‐point increase in reexperiencing symptoms: 1.18; 95% CI: 1.02, 1.36).

**Table 5:** Associations between baseline risk factors, acute symptoms, and persistence of PTSD among participants with acute and persistent diagnoses of PTSD (*N* = 2571).

Predictor	Persistent PTSD		
	Model 1	Model 2	Model 3
Age	1.00 (0.99, 1.01)	1.00 (0.99, 1.01)	1.00 (0.99, 1.01)
Female	1.14 (0.79, 1.65)	1.10 (0.75, 1.59)	1.09 (0.75, 1.59)
Married/living with a partner	1.02 (0.71, 1.46)	1.01 (0.7, 1.45)	1.00 (0.70, 1.44)
< Secondary education	1.21 (0.75, 1.98)	1.26 (0.78, 2.06)	1.23 (0.75, 1.99)
Index trauma: other	1.00 (0.55, 1.82)	1.08 (0.59, 1.99)	1.04 (0.56, 1.9)
Index trauma: assault	0.92 (0.51, 1.67)	0.84 (0.46, 1.53)	0.79 (0.43, 1.46)
Prior non‐interpersonal trauma	1.57 (0.92, 2.66)	1.56 (0.91, 2.67)	1.56 (0.91, 2.67)
Prior interpersonal trauma	1.43 (0.90, 2.28)	1.47 (0.92, 2.35)	1.45 (0.91, 2.33)
MDD symptom severity per 5 point increase	1.17 ^∗∗^ (1.08, 1.27)	1.13 ^∗^ (1.03, 1.24)	1.14 ^∗^ (1.04, 1.25)
PTSD symptom severity per 15 point increase	—	1.14 ^∗^ (1.01, 1.29)	—
PTSD intrusion symptoms per 5 point increase	—	—	1.18 ^∗^ (1.02, 1.36)
PTSD avoidance symptoms per 5 point increase	—	—	0.95 (0.85, 1.06)
PTSD hyperarousal symptoms per 5 point increase	—	—	1.02 (0.87, 1.20)

^∗^ *p* < 0.05.

^∗∗^ *p* < 0.01.

^∗∗∗^ *p* < 0.001.

**Table 6:** Associations between baseline risk factors, acute symptoms, and persistence of MDD among participants with acute and persistent diagnoses of MDD (*N* = 2571).

Predictor	Persistent MDD		
	Model 1	Model 2	Model 3
Age	1.00 (0.98, 1.02)	1.00 (0.98, 1.02)	1.00 (0.98, 1.02)
Female	0.87 (0.58, 1.32)	0.87 (0.58, 1.32)	0.89 (0.59, 1.34)
Married/living with a partner	1.01 (0.63, 1.62)	0.99 (0.62, 1.59)	0.99 (0.62, 1.59)
< Secondary education	1.28 (0.78, 2.1)	1.22 (0.73, 2.03)	1.22 (0.73, 2.03)
Index trauma: other	1.00 (0.51, 1.95)	0.98 (0.5, 1.91)	0.95 (0.48, 1.87)
Index trauma: assault	0.93 (0.45, 1.95)	0.99 (0.47, 2.06)	0.98 (0.46, 2.09)
Prior non‐interpersonal trauma	1.64 (0.88, 3.07)	1.65 (0.88, 3.09)	1.67 (0.89, 3.13)
Prior interpersonal trauma	1.56 (0.86, 2.85)	1.52 (0.83, 2.80)	1.53 (0.83, 2.84)
MDD symptom severity per 5 point increase	–	1.12 (1.00, 1.26)	1.13 ^∗^ (1.00, 1.26)
PTSD symptom severity per 15 point increase	1.15 ^∗^ (1.03, 1.28)	1.08 (0.96, 1.23)	–
PTSD intrusion symptoms per 5 point increase	–	–	1.08 (0.90, 1.30)
PTSD avoidance symptoms per 5 point increase	–	–	1.03 (0.91, 1.16)
PTSD hyperarousal symptoms per 5 point increase	–	–	0.95 (0.79, 1.14)

^∗^ *p* < 0.05.

^∗∗^ *p* < 0.01.

^∗∗∗^ *p* < 0.001.

In models for persistent MDD, acute PTSD symptoms were associated with persistence (Model 1: OR for a 15‐point increase in PTSD symptoms = 1.15; 95% CI: 1.03, 1.28) but after adjustment for acute MDD symptoms neither acute PTSD symptom severity (Model 2) nor acute PTSD symptom cluster severities were significantly associated with persistent MDD (Model 3). Acute MDD symptoms were associated with persistence after adjustment for PTSD symptom cluster severity (Model 3: OR for a 5‐point increase in MDD symptoms: 1.13; 95% CI: 1.00, 1.26).

Results from sensitivity analyses examining the estimated ratio of expected diagnoses of persistent MDD and PTSD cases versus observed (E/O) and Brier scores for the probability of persistent diagnoses compared to observed diagnoses are presented in Table 7. All studies had similar Brier scores across outcomes indicating similar accuracy of prediction models. The E/O was substantially less than 1 in the Hadassah startle study for PTSD indicating that the estimated prevalence of PTSD in the model was severely undercounted. Refitting models predicting persistent versus acute MDD and PTSD excluding Hadassah startle yielded similar results to those presented in Tables 5 and 6 (Supporting Information 1: Table S3). When the persistent period was redefined as 270–456 days since experiencing the index trauma, results were consistent with those presented in Tables 5 and 6 and Figure 1.

**Table 7:** Estimated ratios of the expected number of MDD and PTSD persistent cases and those observed (E/O) in each study and Brier score of the probability of persistent diagnosis compared to actual diagnosis.

Study	Depression		PTSD	
	E/O	Brier score	E/O	Brier score
ASD	1.01	0.21	0.82	0.25
Hadassah startle	0.8	0.22	0.59	0.26
JTOPS	1.04	0.23	1.06	0.23
TCOM	0.88	0.21	1.32	0.21
Amsterdam cortisol	0.96	0.24	0.98	0.23

Abbreviations: ASD, acute stress disorder study; E/O, expected observed ratio; JTOPS, Jerusalem Trauma Outreach and Prevention Study; TCOM, Tachikawa Cohort of Motor Vehicle Accident study.

## 5. Discussion

In this IPD‐MA study of risk factors for acute and persistent PTSD and MDD, there were four major findings. First, PTSD and MDD shared baseline risk factors that increased risk for acute and persistent diagnoses (e.g., female sex, experiencing assaultive trauma) and decreased risk (experiencing an accident versus other trauma types, prior interpersonal and non‐interpersonal trauma). In addition, neither education nor marital status at the time of trauma were associated with either disorder. Second, after adjustment for acute symptoms, no baseline risk factors were associated with persistent PTSD or MDD. Third, while both acute MDD and overall PTSD symptoms were associated with persistent PTSD, when PTSD symptoms were examined by cluster, only intrusion symptoms were associated with persistent PTSD. Finally, only acute MDD symptom severity was associated with persistent MDD after adjustment for all baseline risk factors and PTSD symptom clusters. These findings were robust after removing heterogeneous studies and examining narrower follow‐up times.

This was the first study to systematically examine risk factors for both MDD and PTSD in an emergency room setting with data on both the acute and persistent periods. We found that risk factors for both onset and persistence of each disorder were similar, which indicates that the disorders may share response to trauma [75]. While female sex, index trauma type, and prior trauma were predictive of acute PTSD, once acute symptoms were accounted for no baseline risk factors were significantly associated with persistent PTSD, which supports previous work that well‐established risk factors may increase risk of persistent PTSD through shaping early responses to trauma [76].

Our work further expands on this to show that depressive symptoms following trauma also increase risk for PTSD and that intrusive symptoms are associated with persistence of PTSD. Intrusive symptoms, including intrusive recollections of the events as well as physical and psychological distress from external reminders of the events, are closely related to those of depressive rumination, which is characterized by repeated focus and the replaying of past events or emotions [77]. Engaging in rumination after experiencing a traumatic event has been hypothesized to increase risk for PTSD as it strengthens negative appraisals about the trauma and prevents a complete memory of the trauma from being formed and processed by the individual [78]. Rumination along with depressive and reexperiencing symptoms represent targets for interventions like cognitive behavioral therapy to decrease the odds of symptom persistence following trauma, which suggests that screening for both PTSD and depression would improve targeting of vulnerable individuals following trauma [79]. Future work should examine these symptom‐level mechanisms directly using approaches such as symptom network analysis, which when combined with intensive longitudinal assessments could clarify how depressive rumination and reexperiencing symptoms are connected to other PTSD and MDD symptoms over time and whether they function as bridge symptoms maintaining comorbidity between the disorders [80].

A strength of this study was the large multicultural sample of the ICPP, which included data from four countries and followed individuals from the emergency room through to the persistent period for each disorder. In addition, we performed an IPD‐MA, which allowed us to apply consistent models across all studies and define consistent time periods while benefiting from the larger sample size of the combined studies [81]. In addition we were able to create a harmonized MDD measure across studies by applying IRT to equate PTSD and MDD and pool individual participant data. In our analysis, we used a cutoff score of 13 on the HADS, which equated to a BDI score of 20 indicating moderate depression. A HADS score of 11 or greater has typically been considered the cutoff for probable depression, which we found does not equate to a similar level of distress in our sample [59]. In addition, we were able to create harmonized symptom severities of depressive symptoms using our IRT approach allowing us to examine the association between acute symptom severity and persistent distress.

Our study was also limited by two factors. First, we did not have any measure of PTSD or MDD prior to the index trauma, which has been found to be a consistent predictor of post‐trauma psychopathology [82–85]. Rather than depressive symptoms onsetting after trauma increasing risk for persistent PTSD, depressive symptoms may have existed before the index trauma. Second, we did not have a direct measure of rumination or other personality measures related to MDD or neuroticism that would allow us to further untangle the association between early symptoms and persistent MDD and PTSD. Future studies aimed at predicting persistent symptoms of PTSD and depression should directly assess rumination in the acute period following a traumatic event. Finally, depression was not assessed consistently across all studies requiring the HADS and BDI to be equated across studies. While previous work has shown equating the HADS and BDI to be effective, differences in true measured BDI may exist that could affect these results.

## 6. Conclusions

Our results have implications for future research and practice. First, we have identified several groups presenting in emergency rooms that are at increased risk for developing PTSD and depression both immediately after trauma (acute period) and in the months after (persistent period). Second, we found that acute symptoms, specifically depressive and reexperiencing symptoms, increase risk for the persistent of MDD and PTSD. Individuals reporting substantial symptoms following trauma are at significantly higher risk for persistent PTSD and MDD and could represent an efficacious group to target so that scarce resources may be deployed more effectively [3, 18–20]. Third, the significance of depressive and reexperiencing symptoms following trauma in predicting persistent PTSD, as well as the high observational [7] and genetic [86, 87] correlations between the disorders, may indicate that symptoms of PTSD and depression may represent a single response to trauma rather than two separate disorders. However, this interpretation should be viewed cautiously given that our PTSD diagnoses were based on DSM‐IV criteria and did not incorporate the DSM‐5 negative alterations in cognition and mood cluster [88]. Fourth, the role between baseline risk factors (e.g., female sex or accident type) and acute symptoms should be further explored to understand pathways between acute risk and persistence.

## Author Contributions

Andrew Ratanatharathorn and Karestan C. Koenen were responsible for study conception and design. Richard A. Bryant, Ronald C. Kessler, Karestan C. Koenen, Yutaka Matsuoka, Miranda Olff, and Arieh Y. Shalev contributed to data collection. Andrew Ratanatharathorn, Katherine M. Keyes, Renato Polimanti, Andrew G. Rundle, and Karestan C. Koenen were responsible for analysis and interpretation of results. Andrew Ratanatharathorn created figures and wrote the draft manuscript.

## Acknowledgments

The authors would like to acknowledge Dr. Eugene Laska, who contributed to this work and the International Consortium to Predict PTSD. They thank the participants of the International Consortium to Predict PTSD as well as Paul O’Connor from Nathan Kline Institute for his exceptional work as a steward of the ICPP data.

## Funding

This work was supported by the National Institutes of Mental Health T32 (Grant MH017119) and the International Consortium to Predict PTSD was funded by the US National Institute of Mental Health (Grant MH101227) to Arieh Y. Shalev, Ronald C. Kessler, and Karestan C. Koenen.

## Disclosure

The funder played no role in the study design, data collection, analysis, and interpretation of data or the writing of this manuscript. All authors have reviewed the results and approved the final version of the manuscript.

## Conflicts of Interest

The authors declare no conflicts of interest.

## Supporting Information

Additional supporting information can be found online in the Supporting Information section.

## Supporting information


**Supporting Information** Table S1. DSM‐IV depression symptoms assess by the Beck Depression Inventory II (BDI), Hospital Anxiety and Depression Scale (HADS), and the Clinician Administered PTSD Scale (CAPS). The table notes which question on each scale assesses the specific depression symptom. Table S2. Confirmatory factor results with individual ICPP studies. Table S3. (A) Associations between baseline risk factors, acute symptoms, and persistence of PTSD among participants with acute and persistent diagnoses of PTSD excluding Hadassah Startle. (B) Associations between baseline risk factors, acute symptoms, and persistence of MDD among participants with acute and persistent diagnoses of MDD excluding Hadassah Startle.

## Data Availability

The data that support the findings of this study are available upon request from the corresponding author. The data are not publicly available due to privacy or ethical restrictions.

## References

[1] Benjet C. , Bromet E. , and Karam E. , et al.The Epidemiology of Traumatic Event Exposure Worldwide: Results From the World Mental Health Survey Consortium, Psychological Medicine. (2016) 46, no. 2, 327–343, 10.1017/S0033291715001981, 2-s2.0-84945562504. 26511595 PMC4869975

[2] Koenen K. , Ratanatharathorn A. , and Ng L. , et al.Posttraumatic Stress Disorder in the World Mental Health Surveys, Psychological Medicine. (2017) 47, no. 13, 2260–2274, 10.1017/S0033291717000708, 2-s2.0-85017465989. 28385165 PMC6034513

[3] Rui P. , Kang K. , and Ashman J. , National Hospital Ambulatory Medical Care Survey: 2016 Emergency Department Summary Tables, 2016, U.S. Department of Health and Human Services.

[4] Chiu S. , Niles J. K. , and Webber M. P. , et al.Evaluating Risk Factors and Possible Mediation Effects in Posttraumatic Depression and Posttraumatic Stress Disorder Comorbidity, Public Health Reports. (2011) 126, no. 2, 201–209, 10.1177/003335491112600211, 2-s2.0-79952807291. 21387950 PMC3056033

[5] O’Donnell M. L. , Creamer M. , and Pattison P. , Posttraumatic Stress Disorder and Depression Following Trauma: Understanding Comorbidity, American Journal of Psychiatry. (2004) 161, no. 8, 1390–1396, 10.1176/appi.ajp.161.8.1390, 2-s2.0-3342958864. 15285964

[6] Shalev A. Y. , Freedman S. , and Peri T. , et al.Prospective Study of Posttraumatic Stress Disorder and Depression Following Trauma, American Journal of Psychiatry. (1998) 155, no. 5, 630–637, 10.1176/ajp.155.5.630, 2-s2.0-0031978812. 9585714

[7] Rytwinski N. K. , Scur M. D. , Feeny N. C. , and Youngstrom E. A. , The Co-Occurrence of Major Depressive Disorder among Individuals with Posttraumatic Stress Disorder: A Meta-Analysis, Journal of Traumatic Stress. (2013) 26, no. 3, 299–309, 10.1002/jts.21814, 2-s2.0-84878684268. 23696449

[8] Edmondson D. , Kronish I. M. , Shaffer J. A. , Falzon L. , and Burg M. M. , Posttraumatic Stress Disorder and Risk for Coronary Heart Disease: A Meta-Analytic Review, American Heart Journal. (2013) 166, no. 5, 806–814, 10.1016/j.ahj.2013.07.031, 2-s2.0-84886947053. 24176435 PMC3815706

[9] Van der Kooy K. , Van Hout H. , Marwijk H. , Marten H. , Stehouwer C. , and Beekman A. , Depression and the Risk for Cardiovascular Diseases: Systematic Review and Meta Analysis, International Journal of Geriatric Psychiatry. (2007) 22, no. 7, 613–626, 10.1002/gps.1723, 2-s2.0-34250890185. 17236251

[10] Le Carolyn M. H. , Neylan T. C. , Na B. , Regan M. , Zhang Q. , and Cohen B. E. , Lifetime Trauma Exposure and Prospective Cardiovascular Events and All-cause Mortality: Findings From the Heart and Soul Study, Psychosomatic Medicine. (2013) 75, no. 9, 849–855, 10.1097/PSY.0b013e3182a88846, 2-s2.0-84888131885. 24149074 PMC4014357

[11] Sledjeski E. M. , Speisman B. , and Dierker L. C. , Does Number of Lifetime Traumas Explain the Relationship Between PTSD and Chronic Medical Conditions? Answers From the National Comorbidity Survey-Replication (NCS-R), Journal of Behavioral Medicine. (2008) 31, no. 4, 341–349, 10.1007/s10865-008-9158-3, 2-s2.0-48349086085. 18553129 PMC2659854

[12] Knol M. , Twisk J. W. , Beekman A. T. , Heine R. , Snoek F. J. , and Pouwer F. , Depression as a Risk Factor for the Onset of Type 2 Diabetes Mellitus. A Meta-Analysis, Diabetologia. (2006) 49, no. 5, 837–845, 10.1007/s00125-006-0159-x, 2-s2.0-33646423317. 16520921

[13] Mezuk B. , Eaton W. W. , Albrecht S. , and Golden S. H. , Depression and Type 2 Diabetes Over the Lifespan: A Meta-Analysis, Diabetes Care. (2008) 31, no. 12, 2383–2390, 10.2337/dc08-0985, 2-s2.0-64349113384. 19033418 PMC2584200

[14] Vancampfort D. , Rosenbaum S. , and Ward P. B. , et al.Type 2 Diabetes Among People With Posttraumatic Stress Disorder: Systematic Review and Meta-Analysis, Psychosomatic Medicine. (2016) 78, no. 4, 465–473, 10.1097/PSY.0000000000000297, 2-s2.0-84957837583. 26867081

[15] Chwastiak L. A. , Rosenheck R. A. , Desai R. , and Kazis L. E. , The Association of Psychiatric Illness and All-cause Mortality in the National Department of Veterans Affairs Health Care System, Psychosomatic Medicine. (2010) 72, no. 8, 817–822, 10.1097/PSY.0b013e3181eb33e9, 2-s2.0-78049488466. 20639387 PMC2950891

[16] Cuijpers P. , Vogelzangs N. , Twisk J. , Kleiboer A. , Li J. , and Penninx B. W. , Comprehensive Meta-Analysis of Excess Mortality in Depression in the General Community Versus Patients With Specific Illnesses, American Journal of Psychiatry. (2014) 171, no. 4, 453–462, 10.1176/appi.ajp.2013.13030325, 2-s2.0-84898730774. 24434956

[17] Galatzer-Levy I. R. , Huang S. H. , and Bonanno G. A. , Trajectories of Resilience and Dysfunction Following Potential Trauma: A Review and Statistical Evaluation, Clinical Psychology Review. (2018) 63, 41–55, 10.1016/j.cpr.2018.05.008, 2-s2.0-85048240971. 29902711

[18] Gartlehner G. , Wagner G. , and Matyas N. , et al.Pharmacological and Non-Pharmacological Treatments for Major Depressive Disorder: Review of Systematic Reviews, BMJ Open. (2017) 7, no. 6, 10.1136/bmjopen-2016-014912, 2-s2.0-85020550158. PMC5623437 28615268

[19] Lee D. J. , Schnitzlein C. W. , Wolf J. P. , Vythilingam M. , Rasmusson A. M. , and Hoge C. W. , Psychotherapy Versus Pharmacotherapy for Posttraumatic Stress Disorder: Systemic Review and Meta-Analyses to Determine First-Line Treatments, Depression and Anxiety. (2016) 33, no. 9, 792–806, 10.1002/da.22511, 2-s2.0-84985943126. 27126398

[20] Farah W. H. , Alsawas M. , and Mainou M. , et al.Non-Pharmacological Treatment of Depression: A Systematic Review and Evidence Map, BMJ Evidence-Based Medicine. (2016) 21, no. 6, 214–221, 10.1136/ebmed-2016-110522, 2-s2.0-84995443078. 27836921

[21] Shalev A. Y. , Ankri Y. , and Gilad M. , et al.Long-Term Outcome of Early Interventions to Prevent Posttraumatic Stress Disorder, The Journal of Clinical Psychiatry. (2016) 77, no. 5, e580–e587, 10.4088/JCP.15m09932, 2-s2.0-84973474331. 27135249

[22] Shalev A. Y. , Ankri Y. , Israeli-Shalev Y. , Peleg T. , Adessky R. , and Freedman S. , Prevention of Posttraumatic Stress Disorder by Early Treatment: Results From the Jerusalem Trauma Outreach and Prevention Study, Archives of General Psychiatry. (2012) 69, no. 2, 166–176, 10.1001/archgenpsychiatry.2011.127, 2-s2.0-84856738584. 21969418

[23] Jonas D. E. , Cusack K. , and Forneris C. A. , et al.Psychological and Pharmacological Treatments for Adults with Posttraumatic Stress Disorder (PTSD), 2013, Agency for Healthcare Research and Quality (US). 23658937

[24] Brewin C. R. , Andrews B. , and Valentine J. D. , Meta-Analysis of Risk Factors for Posttraumatic Stress Disorder in Trauma-Exposed Adults, 2000, American Psychological Association. 10.1037//0022-006x.68.5.748 11068961

[25] Nanni V. , Uher R. , and Danese A. , Childhood Maltreatment Predicts Unfavorable Course of Illness and Treatment Outcome in Depression: A Meta-Analysis, American Journal of Psychiatry. (2012) 169, no. 2, 141–151, 10.1176/appi.ajp.2011.11020335, 2-s2.0-84857582541. 22420036

[26] Koenen K. C. , Stellman J. M. , Stellman S. D. , and Sommer J. F.Jr, Risk Factors for Course of Posttraumatic Stress Disorder Among Vietnam Veterans: A 14-Year Follow-Up of American Legionnaires, Journal of Consulting & Clinical Psychology. (2003) 71, no. 6, 980–986, 10.1037/0022-006X.71.6.980, 2-s2.0-0344064334. 14622073

[27] Lorant V. , Deliège D. , Eaton W. , Robert A. , Philippot P. , and Ansseau M. , Socioeconomic Inequalities in Depression: A Meta-Analysis, American Journal of Epidemiology. (2003) 157, no. 2, 98–112, 10.1093/aje/kwf182, 2-s2.0-0037438651. 12522017

[28] Mollica R. F. , Sarajlić N. , Chernoff M. , Lavelle J. , Vuković I. S. , and Massagli M. P. , Longitudinal Study of Psychiatric Symptoms, Disability, Mortality, and Emigration Among Bosnian Refugees, Journal of the American Medical Association. (2001) 286, no. 5, 546–554, 10.1001/jama.286.5.546, 2-s2.0-0035413910. 11476656

[29] Silver R. C. , Holman E. A. , McIntosh D. N. , Poulin M. , and Gil-Rivas V. , Nationwide Longitudinal Study of Psychological Responses to September 11, JAMA. (2002) 288, no. 10, 1235–1244, 10.1001/jama.288.10.1235, 2-s2.0-0037063403. 12215130

[30] Kessler R. C. , Epidemiology of Women and Depression, Journal of Affective Disorders. (2003) 74, no. 1, 5–13, 10.1016/S0165-0327(02)00426-3, 2-s2.0-0037341830. 12646294

[31] Eaton W. W. , Shao H. , Nestadt G. , Lee B. H. , Bienvenu O. J. , and Zandi P. , Population-Based Study of First Onset and Chronicity in Major Depressive Disorder, Archives of General Psychiatry. (2008) 65, no. 5, 513–520, 10.1001/archpsyc.65.5.513, 2-s2.0-43149091691. 18458203 PMC2761826

[32] Moffitt T. E. , Harrington H. , and Caspi A. , et al.Depression and Generalized Anxiety Disorder: Cumulative and Sequential Comorbidity in a Birth Cohort Followed Prospectively to Age 32 Years, Archives of General Psychiatry. (2007) 64, no. 6, 651–660, 10.1001/archpsyc.64.6.651, 2-s2.0-34249948211. 17548747

[33] Moffitt T. E. , Caspi A. , and Taylor A. , et al.How Common Are Common Mental Disorders? Evidence That Lifetime Prevalence Rates Are Doubled by Prospective Versus Retrospective Ascertainment, Psychological Medicine. (2010) 40, no. 6, 899–909, 10.1017/S0033291709991036, 2-s2.0-77952428781. 19719899 PMC3572710

[34] Qi W. , Ratanatharathorn A. , and Gevonden M. , et al.Application of Data Pooling to Longitudinal Studies of Early Post-Traumatic Stress Disorder (PTSD): The International Consortium to Predict PTSD (ICPP) Project, European Journal of Psychotraumatology. (2018) 9, no. 1, 10.1080/20008198.2018.1476442. PMC6008580 29938009

[35] Stewart L. A. , Clarke M. , and Rovers M. , et al.Preferred Reporting Items for a Systematic Review and Meta-Analysis of Individual Participant Data: The PRISMA-IPD Statement, Journal of the American Medical Association. (2015) 313, no. 16, 1657–1665, 10.1001/jama.2015.3656, 2-s2.0-84928753669. 25919529

[36] Hussong A. M. , Curran P. J. , and Bauer D. J. , Integrative Data Analysis in Clinical Psychology Research, Annual Review of Clinical Psychology. (2013) 9, no. 1, 61–89, 10.1146/annurev-clinpsy-050212-185522, 2-s2.0-84875889078. PMC3924786 23394226

[37] Jolani S. , Debray T. P. , Koffijberg H. , van Buuren S. , and Moons K. G. , Imputation of Systematically Missing Predictors in an Individual Participant Data Meta-Analysis: A Generalized Approach Using MICE, Statistics in Medicine. (2015) 34, no. 11, 1841–1863, 10.1002/sim.6451, 2-s2.0-84926444242. 25663182

[38] Kunkel D. and Kaizar E. E. , A Comparison of Existing Methods for Multiple Imputation in Individual Participant Data Meta-Analysis, Statistics in Medicine. (2017) 36, no. 22, 3507–3532, 10.1002/sim.7388, 2-s2.0-85022323535. 28695667 PMC5582994

[39] Debray T. P. , Moons K. G. , Ahmed I. , Koffijberg H. , and Riley R. D. , A Framework for Developing, Implementing, and Evaluating Clinical Prediction Models in an Individual Participant Data Meta-Analysis, Statistics in Medicine. (2013) 32, no. 18, 3158–3180, 10.1002/sim.5732, 2-s2.0-84880044696. 23307585

[40] Shalev A. Y. , Gevonden M. , and Ratanatharathorn A. , et al.Estimating the Risk of PTSD in Recent Trauma Survivors: Results of the International Consortium to Predict PTSD (ICPP), World Psychiatry. (2019) 18, no. 1, 77–87, 10.1002/wps.20608, 2-s2.0-85059422925. 30600620 PMC6313248

[41] King D. W. , King L. A. , McArdle J. J. , Shalev A. Y. , and Doron-LaMarca S. , Sequential Temporal Dependencies in Associations between Symptoms of Depression and Posttraumatic Stress Disorder: An Application of Bivariate Latent Difference Score Structural Equation Modeling, Multivariate Behavioral Research. (2009) 44, no. 4, 437–464, 10.1080/00273170903103308, 2-s2.0-77951653318. 26735592

[42] Bryant R. A. , Creamer M. , O’Donnell M. L. , Silove D. , and McFarlane A. C. , A Multisite Study of the Capacity of Acute Stress Disorder Diagnosis to Predict Posttraumatic Stress Disorder, Journal of Clinical Psychiatry. (2008) 69, no. 6, 923–929, 10.4088/JCP.v69n0606, 2-s2.0-46749090584. 18422396

[43] Shalev A. Y. , Peri T. , Brandes D. , Freedman S. , Orr S. P. , and Pitman R. K. , Auditory Startle Response in Trauma Survivors with Posttraumatic Stress Disorder: A Prospective Study, American Journal of Psychiatry. (2000) 157, no. 2, 255–261, 10.1176/appi.ajp.157.2.255, 2-s2.0-0033953439. 10671396

[44] Matsuoka Y. , Nishi D. , and Nakajima S. , et al.The Tachikawa Cohort of Motor Vehicle Accident Study Investigating Psychological Distress: Design, Methods and Cohort Profiles, Social Psychiatry & Psychiatric Epidemiology. (2009) 44, no. 4, 333–340, 10.1007/s00127-008-0438-6, 2-s2.0-63949087317. 18818856

[45] Mouthaan J. , Sijbrandij M. , Luitse J. S. , Goslings J. C. , Gersons B. P. , and Olff M. , The Role of Acute Cortisol and DHEAS in Predicting Acute and Chronic PTSD Symptoms, Psychoneuroendocrinology. (2014) 45, 179–186, 10.1016/j.psyneuen.2014.04.001, 2-s2.0-84901199183. 24845188

[46] Matsuoka Y. , Nishi D. , Nakajima S. , Kim Y. , Homma M. , and Otomo Y. , Incidence and Prediction of Psychiatric Morbidity After a Motor Vehicle Accident in Japan: The Tachikawa Cohort of Motor Vehicle Accident Study, Critical Care Medicine. (2008) 36, no. 1, 74–80, 10.1097/01.CCM.0000291650.70816.D6, 2-s2.0-37549045994. 18090377

[47] Blake D. D. , Weathers F. W. , and Nagy L. M. , et al.The Development of a Clinician-Administered PTSD Scale, Journal of Traumatic Stress. (1995) 8, no. 1, 75–90. 7712061 10.1007/BF02105408

[48] Weathers F. W. , Keane T. M. , and Davidson J. R. , Clinician-Administered PTSD Scale: A Review of the First Ten Years of Research, Depression and Anxiety. (2001) 13, no. 3, 132–156, 10.1002/da.1029, 2-s2.0-0035006807. 11387733

[49] American Psychiatric Association , Diagnostic and Statistical Manual, 1994, 4th edition, American Psychiatric Association.

[50] Weathers F. W. , Ruscio A. M. , and Keane T. M. , Psychometric Properties of Nine Scoring Rules for the Clinician-Administered Posttraumatic Stress Disorder Scale, Psychological Assessment. (1999) 11, no. 2, 124–133, 10.1037/1040-3590.11.2.124, 2-s2.0-0033037116.

[51] Shalev A. , Ratanatharathorn A. , and Qi W. , et al.Robust Prediction of PTSD Likelihood From Early Symptoms Results From the International Consortium to Predict PTSD (ICPP) Pooled Data Analysis, 2017, Nature Publishing Group Macmillan Building, S123–S124.

[52] Beck A. T. , Steer R. A. , and Brown G. K. , Beck Depression Inventory-II, Health. (1996) 78, no. 2, 490–498.

[53] Zigmond A. S. and Snaith R. P. , The Hospital Anxiety and Depression Scale, Acta Psychiatrica Scandinavica. (1983) 67, no. 6, 361–370, 10.1111/j.1600-0447.1983.tb09716.x, 2-s2.0-0020527558. 6880820

[54] Johnston M. , Pollard B. , and Hennessey P. , Construct Validation of the Hospital Anxiety and Depression Scale With Clinical Populations, Journal of Psychosomatic Research. (2000) 48, no. 6, 579–584, 10.1016/S0022-3999(00)00102-1, 2-s2.0-0033819407. 11033377

[55] Claxton J. , Vibhakar V. , Allen L. , Finn J. , Gee B. , and Meiser-Stedman R. , Risk Factors for Depression in Trauma-Exposed Children and Adolescents: A Systematic Review and Meta-Analysis, Journal of Affective Disorders Reports. (2021) 5, 10.1016/j.jadr.2021.100150. 31203106

[56] Tang B. , Liu X. , Liu Y. , Xue C. , and Zhang L. , A Meta-Analysis of Risk Factors for Depression in Adults and Children After Natural Disasters, BMC Public Health. (2014) 14, no. 1, 1–12, 10.1186/1471-2458-14-623, 2-s2.0-84902702385. 24941890 PMC4077641

[57] McQuaid J. R. , Pedrelli P. , McCahill M. , and Stein M. B. , Reported Trauma, Post-Traumatic Stress Disorder and Major Depression among Primary Care Patients, Psychological Medicine. (2001) 31, no. 7, 1249–1257, 10.1017/s0033291701004202, 2-s2.0-0034763291. 11681551

[58] Fowler J. C. , Allen J. G. , Oldham J. M. , and Frueh B. C. , Exposure to Interpersonal Trauma, Attachment Insecurity, and Depression Severity, Journal of Affective Disorders. (2013) 149, no. 1–3, 313–318, 10.1016/j.jad.2013.01.045, 2-s2.0-84878489388. 23507367

[59] Smarr K. L. and Keefer A. L. , Measures of Depression and Depressive Symptoms: Beck Depression Inventory-II (BDI-II), Center for Epidemiologic Studies Depression Scale (CES-D), Geriatric Depression Scale (GDS), Hospital Anxiety and Depression Scale (HADS), and Patient Health Questionnaire-9 (PHQ-9), Arthritis Care and Research. (2011) 63, no. S11, 10.1002/acr.20556, 2-s2.0-80755127057. 22588766

[60] Dozois D. J. , Dobson K. S. , and Ahnberg J. L. , A Psychometric Evaluation of the Beck Depression Inventory–II, Psychological Assessment. (1998) 10, no. 2.

[61] Storch E. A. , Roberti J. W. , and Roth D. A. , Factor Structure, Concurrent Validity, and Internal Consistency of the Beck Depression Inventory—Second Edition in a Sample of College Students, Depression & Anxiety. (2004) 19, no. 3, 187–189, 10.1002/da.20002, 2-s2.0-2542478006. 15129421

[62] Bjelland I. , Dahl A. A. , Haug T. T. , and Neckelmann D. , The Validity of the Hospital Anxiety and Depression Scale: An Updated Literature Review, Journal of Psychosomatic Research. (2002) 52, no. 2, 69–77, 10.1016/S0022-3999(01)00296-3, 2-s2.0-0036188619. 11832252

[63] Kolen M. J. and Brennan R. L. , Test Equating, Scaling, and Linking, 2004, Springer New York.

[64] Lt H. and Bentler P. M. , Cutoff Criteria for Fit Indexes in Covariance Structure Analysis: Conventional Criteria Versus New Alternatives, Structural Equation Modeling: A Multidisciplinary Journal. (1999) 6, no. 1, 1–55.

[65] West S. G. , Taylor A. B. , and Wu W. , Model Fit and Model Selection in Structural Equation Modeling, Handbook of Structural Equation Modeling. (2012) 1, 209–231.

[66] Browne M. W. , Alternative Ways of Assessing Model Fit, Testing Structural Equation Models. (1993) 136–162.

[67] Elhai J. D. , Contractor A. A. , and Tamburrino M. , et al.The Factor Structure of Major Depression Symptoms: A Test of Four Competing Models Using the Patient Health Questionnaire-9, Psychiatry Research. (2012) 199, no. 3, 169–173, 10.1016/j.psychres.2012.05.018, 2-s2.0-84869886660. 22698261

[68] Samejima F. , Graded Response Models. Handbook of Item Response Theory, Volume One, 2016, Chapman and Hall/CRC, 123–136.

[69] Marco G. L. , Item Characteristic Curve Solutions to Three Intractable Testing Problems 1, ETS Research Bulletin Series. (1977) 1977, no. 1, i–41, 10.1002/j.2333-8504.1977.tb01136.x.

[70] Buuren S. and mice G.-O. K. , Multivariate Imputation by Chained Equations in R, Journal of Statistical Software. (2011) 45, no. 3, 10.18637/jss.v045.i03.

[71] Quartagno M. and Carpenter J. , Multiple Imputation for IPD Meta-Analysis: Allowing for Heterogeneity and Studies with Missing Covariates, Statistics in Medicine. (2016) 35, no. 17, 2938–2954, 10.1002/sim.6837, 2-s2.0-84977572868. 26681666 PMC5064632

[72] Rubin D. B. , Multiple Imputation for Nonresponse in Surveys, 2004, John Wiley & Sons.

[73] Gelman A. and Stern H. , The Difference Between “Significant” and “Not Significant” Is Not Itself Statistically Significant, The American Statistician. (2006) 60, no. 4, 328–331, 10.1198/000313006X152649, 2-s2.0-33750898613.

[74] Brier G. W. , Verification of Forecasts Expressed in Terms of Probability, Monthey Weather Review. (1950) 78, no. 1, 1–3, 10.1175/1520-0493(1950)078<0001:VOFEIT>2.0.CO;2.

[75] Flory J. D. and Yehuda R. , Comorbidity between Post-Traumatic Stress Disorder and Major Depressive Disorder: Alternative Explanations and Treatment Considerations, Dialogues in Clinical Neuroscience. (2015) 17, no. 2, 141–150, 10.31887/DCNS.2015.17.2/jflory. 26246789 PMC4518698

[76] Shalev A. Y. and Freedman S. , PTSD Following Terrorist Attacks: A Prospective Evaluation, American Journal of Psychiatry. (2005) 162, no. 6, 1188–1191, 10.1176/appi.ajp.162.6.1188, 2-s2.0-20044375593. 15930068

[77] Moulds M. L. , Bisby M. A. , Wild J. , and Bryant R. A. , Rumination in Posttraumatic Stress Disorder: A Systematic Review, Clinical Psychology Review. (2020) 82, 10.1016/j.cpr.2020.101910. 32971312

[78] Ehlers A. and Clark D. M. , A Cognitive Model of Posttraumatic Stress Disorder, Behaviour Research & Therapy. (2000) 38, no. 4, 319–345, 10.1016/S0005-7967(99)00123-0, 2-s2.0-0033960841. 10761279

[79] Schumm H. , Krüger-Gottschalk A. , and Dyer A. , et al.Mechanisms of Change in Trauma-Focused Treatment for PTSD: The Role of Rumination, Behaviour Research and Therapy. (2022) 148, 10.1016/j.brat.2021.104009. 34823161

[80] Choi K. W. , Batchelder A. W. , Ehlinger P. P. , Safren S. A. , and O’Cleirigh C. , Applying Network Analysis to Psychological Comorbidity and Health Behavior: Depression, PTSD, and Sexual Risk in Sexual Minority Men With Trauma Histories, Journal of Consulting and Clinical Psychology. (2017) 85, no. 12, 1158–1170, 10.1037/ccp0000241, 2-s2.0-85035811124. 29189032 PMC5724394

[81] Riley R. D. , Lambert P. C. , and Abo-Zaid G. , Meta-Analysis of Individual Participant Data: Rationale, Conduct, and Reporting, BMJ. (2010) 340, c221–c221, 10.1136/bmj.c221, 2-s2.0-77749306261. 20139215

[82] Sayed S. , Iacoviello B. M. , and Charney D. S. , Risk Factors for the Development of Psychopathology Following Trauma, Current Psychiatry Reports. (2015) 17, 1–7. 26206108 10.1007/s11920-015-0612-y

[83] Parslow R. A. , Jorm A. F. , and Christensen H. , Associations of Pre-Trauma Attributes and Trauma Exposure With Screening Positive for PTSD: Analysis of a Community-Based Study of 2085 Young Adults, Psychological Medicine. (2006) 36, no. 3, 387–395, 10.1017/S0033291705006306, 2-s2.0-33646573679. 16255836

[84] Harnett N. , Dumornay N. , and Delity M. , et al.Prior Differences in Previous Trauma Exposure Primarily Drive the Observed Racial/Ethnic Differences in Posttrauma Depression and Anxiety Following a Recent Trauma, Psychological Medicine. (2023) 53, no. 6, 1–2562, 10.1017/S0033291721004475. 35094717 PMC9339026

[85] Breslau N. , Peterson E. L. , and Schultz L. R. , A Second Look at Prior Trauma and the Posttraumatic Stress Disorder Effects of Subsequent Trauma: A Prospective Epidemiological Study, Archives of General Psychiatry. (2008) 65, no. 4, 431–437, 10.1001/archpsyc.65.4.431, 2-s2.0-42049122131. 18391131

[86] Mundy J. , Hübel C. , and Gelernter J. , et al.Psychological Trauma and the Genetic Overlap Between Posttraumatic Stress Disorder and Major Depressive Disorder, Psychological Medicine. (2022) 52, no. 16, 3975–3984, 10.1017/S0033291721000830. 34085609 PMC8962503

[87] Nievergelt C. M. , Maihofer A. X. , and Klengel T. , et al.International Meta-Analysis of PTSD Genome-Wide Association Studies Identifies Sex-and Ancestry-Specific Genetic Risk Loci, Nature Communications. (2019) 10, no. 1, 10.1038/s41467-019-12576-w, 2-s2.0-85073062147. PMC6783435 31594949

[88] Edition F. , Diagnostic and Statistical Manual of Mental Disorders, American Psychiatric Association. (2013) 21, no. 21, 591–643.

