# Supplementary material for: Individual Participant Data Meta‐Analysis Identifies Risk Factors for Acute and Persistent Posttraumatic Stress Disorder and Depression Symptoms Following Trauma
Source: Depress Anxiety. 2026 May 4;2026:9859948. doi: 10.1155/da/9859948 (PMC13138216; doi:10.1155/da/9859948)
Supplement: Supplementary file 1 — Supporting Information Table S1. DSM‐IV depression symptoms assess by the Beck Depression Inventory II (BDI), Hospital Anxiety and Depression Scale (HADS), and the Clinician Administered PTSD Scale (CAPS). The table notes which question on each scale assesses the specific depression symptom. Table S2. Confirmatory factor results with individual ICPP studies. Table S3. (A) Associations between baseline risk factors, acute symptoms, and persistence of PTSD among participants with acute and persistent diagnoses of PTSD excluding Hadassah Startle. (B) Associations between baseline risk factors, acute symptoms, and persistence of MDD among participants with acute and persistent diagnoses of MDD excluding Hadassah Startle. [file DA-2026-9859948-s001.docx]

Supplemental Table 1: DSM-IV depression symptoms assess by the Beck Depression Inventory II (BDI), Hospital Anxiety and Depression Scale (HADS), and the Clinician Administered PTSD Scale (CAPS). The table notes which question on each scale assesses the specific depression symptom.

| **Depression Symptoms** | **Depression instruments** | | **Anchor items** |
| --- | --- | --- | --- |
|  | **BDI** | **HADS** | **CAPS** |
| 1. Depressed mood most of the day. | 1, 10, 17 | 4, 6, 14 |  |
| 2. Diminished interest or pleasure in all or most activities. | 4, 12 | 2, 12 | C4 |
| 3. Significant unintentional weight loss or gain. | 18 |  |  |
| 4. Insomnia or sleeping too much. | 16 |  | D1 |
| 5. Agitation or psychomotor retardation noticed by others. | 11 | 8 |  |
| 6. Fatigue or loss of energy. | 15, 20 |  |  |
| 7. Feelings of worthlessness or excessive guilt. | 5, 6, 7, 8, 14 | 10 |  |
| 8. Diminished ability to think or concentrate, or indecisiveness. | 13, 19 |  | D3 |
| 9. Recurrent thoughts of death | 9 |  |  |

Supplemental Table 2: Confirmatory Factor Results with individual ICPP studies

| **Study** | **Instrument** | **Factors** | **CFI** | **TLI** | **RMSEA** |
| --- | --- | --- | --- | --- | --- |
| ASD | HADS | One | 0.93 | 0.91 | 0.07 |
| Hadassah Startle | BDI | One | 0.77 | 0.74 | 0.1 |
| JTOPS | BDI | One | 0.83 | 0.81 | 0.08 |
| TCOM | HADS | One | 0.89 | 0.86 | 0.11 |
| Amsterdam Cortisol | HADS | One | 0.94 | 0.92 | 0.09 |
| Pooled ICPP | HADS | One | 0.99 | 0.98 | 0.06 |
| Pooled ICPP | BDI | One | 0.98 | 0.98 | 0.07 |
| Pooled ICPP | HADS | Two | 0.99 | 0.98 | 0.05 |
| Pooled ICPP | BDI | Two | 0.99 | 0.98 | 0.06 |

Abbreviations: ASD = Acute Stress Disorder; JTOPS = Jerusalem Trauma Outreach and Prevention Study; TCOM = Tachikawa Cohort of Motor Vehicle Accident study; PTSD = Posttraumatic Stress Disorder; MDD = Major Depressive Disorder; HADS = Hospital Anxiety and Depression Scale; BDI = Beck Depression Inventory II ; ICPP = International Consortium to Predict PTSD; CFI = comparative fit index, TLI = Tucker Lew Index, RMSEA = root mean square error of approximation.

**Supplementary Table 3A: Associations between baseline risk factors, acute symptoms, and persistence of PTSD among participants with acute and persistent diagnoses of PTSD excluding Hadassah Startle.**

|  | **Persistent PTSD** | | |
| --- | --- | --- | --- |
| **Predictor** | **Model 1** | **Model 2** | **Model 3** |
| Age | 1 (0.99, 1.02) | 1 (0.99, 1.02) | 1 (0.99, 1.02) |
| Female | 1.19 (0.81, 1.75) | 1.14 (0.78, 1.69) | 1.15 (0.78, 1.69) |
| Married / living with a partner | 1.02 (0.7, 1.48) | 1.02 (0.7, 1.47) | 1.01 (0.7, 1.46) |
| < Secondary Education | 1.29 (0.8, 2.08) | 1.33 (0.83, 2.16) | 1.3 (0.8, 2.09) |
| Index trauma: other | 1 (0.54, 1.85) | 1.07 (0.57, 2.01) | 1.03 (0.55, 1.92) |
| Index trauma: assault | 0.93 (0.5, 1.7) | 0.85 (0.46, 1.57) | 0.8 (0.43, 1.5) |
| Prior non-interpersonal trauma | 1.52 (0.88, 2.64) | 1.52 (0.87, 2.65) | 1.52 (0.86, 2.66) |
| Prior interpersonal trauma | 1.44 (0.89, 2.34) | 1.48 (0.91, 2.4) | 1.46 (0.9, 2.39) |
| MDD symptom severity per 5 point increase | 1.03** (1.01, 1.05) | 1.02* (1, 1.04) | 1.03* (1.01, 1.05) |
| PTSD symptom severity per 15 point increase | - | 1.01 (1, 1.02) | - |
| PTSD intrusion symptoms per 5 point increase | - | - | 1.03* (1, 1.06) |
| PTSD avoidance symptoms per 5 point increase | - | - | 0.99 (0.97, 1.01) |
| PTSD hyperarousal symptoms per 5 point increase | - | - | 1 (0.97, 1.04) |

**Supplementary Table 3B: Associations between baseline risk factors, acute symptoms, and persistence of MDD among participants with acute and persistent diagnoses of MDD excluding Hadassah Startle.**

|  | **Persistent MDD** | | |
| --- | --- | --- | --- |
| **Predictor** | **Model 1** | **Model 2** | **Model 3** |
| Age | 1 (0.98, 1.02) | 1 (0.98, 1.02) | 1 (0.98, 1.02) |
| Female | 0.85 (0.56, 1.3) | 0.85 (0.56, 1.3) | 0.86 (0.56, 1.32) |
| Married / living with a partner | 1.06 (0.65, 1.72) | 1.04 (0.64, 1.68) | 1.04 (0.64, 1.69) |
| < Secondary Education | 1.29 (0.79, 2.13) | 1.23 (0.74, 2.05) | 1.23 (0.74, 2.06) |
| Index trauma: other | 1.02 (0.52, 2) | 0.99 (0.5, 1.94) | 0.96 (0.49, 1.9) |
| Index trauma: assault | 0.95 (0.44, 2.04) | 1 (0.46, 2.17) | 1 (0.44, 2.23) |
| Prior non-interpersonal trauma | 1.6 (0.85, 3.04) | 1.63 (0.86, 3.08) | 1.64 (0.87, 3.09) |
| Prior interpersonal trauma | 1.56 (0.84, 2.89) | 1.52 (0.82, 2.84) | 1.53 (0.81, 2.86) |
| MDD symptom severity per 5 point increase | - | 1.02* (1, 1.05) | 1.02* (1, 1.05) |
| PTSD symptom severity per 15 point increase | 1.01* (1, 1.02) | 1 (1, 1.01) | - |
| PTSD intrusion symptoms per 5 point increase | - | - | 1.01 (0.98, 1.05) |
| PTSD avoidance symptoms per 5 point increase | - | - | 1 (0.98, 1.03) |
| PTSD hyperarousal symptoms per 5 point increase | - | - | 0.99 (0.95, 1.03) |
